# Revision of Acetabular Component with a Debonded Porous Coating in an Elderly Patient

**DOI:** 10.3390/geriatrics5040073

**Published:** 2020-10-09

**Authors:** Katarina Barbaric Starcevic, Goran Bicanic, Stjepan Dokuzovic, Damir Starcevic, Domagoj Delimar

**Affiliations:** 1 Department of Orthopaedic Surgery, University Hospital Centre Zagreb, Salata 6, 10000 Zagreb, Croatia; katarina.barbaric@hotmail.com (K.B.S.); domagoj.delimar@kbc-zagreb.hr (D.D.); 2 Al Zahra Hospital Dubai, Department of Orthopaedic Surgery, 00000 Dubai, UAE; goran@bicanic.eu; 3 Clinic for Traumatology, University Hospital Centre Sisters of Charity, Draškovićeva 19, 10000 Zagreb, Croatia; s.dokuzovic07@gmail.com; 4 Special Hospital for orthopedic surgery “Akromion”, Ljudevita Gaja 2, 49217 Krapinske Toplice, Croatia; 5 School of Medicine, University of Zagreb, Salata 6, 10000 Zagreb, Croatia

**Keywords:** revision, total hip arthroplasty, debonding, acetabular component, porous coating, elderly patient

## Abstract

Debonding of the porous coating from the acetabular component of a total hip endoprosthesis is a rare complication. Revision total hip arthroplasty for an unstable acetabular component with a debonded porous coating strongly fixed to the bone can be challenging, especially in elderly patients of poor overall health. In such patients, revision procedures should be as simple and safe as possible. We present our technique of solving that problem in a case of an 82-year-old female with bad general condition and unstable acetabular component of hip endoprosthesis. Because of extremely deficient bone stock, a well-fixed porous coating was left in acetabulum to serve as a “cage“, allowing cemented acetabular component placement. This procedure can reduce the risk of intraoperative bone fracture, blood loss, and duration of surgery, which is important in elderly patients with poor overall health.

## 1. Introduction

Revision total hip arthroplasty (THA) in elderly patients with many comorbidities presents a significant challenge for the orthopedic surgeon. In such cases, a clear assessment of a patient’s ability to endure surgical treatment is required. In addition, it is necessary to perform as simple a procedure as possible, which will provide a good result with minimum perioperative risk. Different technical complications during revision surgeries are possible and have been described in the literature [1].

A rare complication is debonding of the porous coating of the acetabular component of a total hip endoprosthesis [2,3,4]. Porous coating of uncemented implants are in use to obtain biological fixation and improve longevity of total hip endoprosthesis by allowing bone ingrowth within porous surfaces [5]. Today there are different materials and types of porous coating in use. There are only two papers published reporting three cases about debonding of porous coating in Biomet Romanus cup, one case 3 years, one 5 years, and one 10 years after implantation [2,4]. In our patient, the total hip endoprosthesis with Biomet Romanus cup was implanted 24 years ago.

In patients in good general health, revision surgery for this complication involves removal of the debonded layer, exposing good acetabular bone stock, and replacement with a new acetabular component. In a patient with poor overall health, with a higher risk of intraoperative blood loss and extremely poor bone stock, removal of a debonded porous coating presents an additional challenge. We present our technique of revision THA in an elderly patient with a deboned porous coating of the acetabular cup.

## 2. Case Presentation

The patient gave her written informed consent for presentation and reporting her case in scientific literature. The aim and methodology in the manuscript are in accordance with the Helsinki declaration and ethical standards for publication and taking into account that written permission of the patient has been received.

In February 2018, an 82-year-old female came to our clinic presenting with total hip endoprosthesis luxation. She had THA on her left hip 24 years ago. An uncemented Biomet (Berlin, Germany) total hip endoprosthesis with Romanus cup was used. She has done well and had no problems with the operated hip for 18 years after her initial surgery. In May 2012, she fell and had a periprosthetic fracture of her left femur. She was operated on in our clinic using a revision femoral stem, while the acetabular component was found to be stable both radiographically and intraoperatively. Afterwards, the patient experienced hip dislocations on two occasions caused by falling on the ground (one in a 2015 and the second in 2017). Both times she was treated with closed reduction and six weeks of immobilization in a hip abduction orthosis. Finally, in February of 2018, a third luxation occurred, without trauma, while sitting on a chair.

Physical examination upon admission revealed pain in her left hip with manipulation of her leg. She was unable to stand or walk. Her left leg was shortened and fixed in external rotation. Blood exams showed mild anemia with hemoglobin value of 112 g/L. Radiograms of the acetabular component of the hip endoprosthesis showed signs of instability, while the femoral component was found stable. In addition, the patient was of asthenic constitution with numerous comorbidities, most significant of which were hypertension, hypothyroidism, obstructive pulmonary disease, and extreme osteoporosis. She was classified as ASA III for her physical status [6].

**Figure 1:**
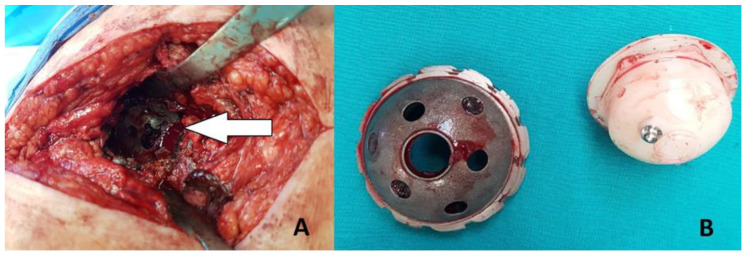
(**A**) Intraoperative photograph showing the porous coating of the acetabular component well-fixed in the acetabulum along its whole surface (white arrow). (**B**) Removed acetabular component devoid of its porous coating and the corresponding polyethylene insert.

Revision surgery in the form of reacetabuloplasty was indicated. Due to the patient’s weak general health, surgical treatment posed an increased perioperative risk. In addition, possible major blood loss during revision surgery had to be addressed. Therefore, we decided to perform as simple and safe procedure as possible to minimize possible perioperative complications. After preoperative preparation, on 8 February 2018, the surgical procedure was performed in spinal anesthesia in the lateral decubitus position. A direct lateral approach was used to expose the left hip endoprosthesis. There was noticeable deterioration of the polyethylene insert, which was removed revealing absolute instability of the Biomet Romanus acetabular component. There were no signs of synovitis and metallosis. After effortless removal of the acetabular component, the porous coating was found completely detached from the acetabular component and still fixed to the acetabulum. It was intact along its whole circumference and firmly attached to the bone (Figure 1). Extremely osteoporotic acetabular bone stock was also observed. There was no anterior bone wall, and only a small amount of bone wall was found posteriorly on the acetabulum. Medially, palpation with the instrument through the porous coating revealed that there was only a thin cortical wall (Paprosky type 3A) [7]. The porous coating was firmly fixed on acetabular bone, and removing it would cause removal of some amount of the bone.

Due to the patient’s poor general condition and really poor acetabular bone stock, we decided to leave the porous coating fixed in the acetabulum in order to reduce the chance of intraoperative fracture, protrusion into the pelvis, and blood loss. It also served as a sort of a “cage”, which facilitated safe placement of the new cemented acetabular component. Retention holes for bone cement were drilled in the porous coating layer, and a cemented acetabular component (size 46) was inserted in the usual manner. After checking the stability of the acetabular component, the femoral revision stem was also tested. The stem was stable; however, the femoral head prosthesis was replaced with one with a longer neck to provide additional stability to the hip. After reduction, provocative tests confirmed hip endoprosthesis stability. The wound was closed in the usual manner by suturing the abductor muscles and fascia lata, ending with subcutis and skin closure.

**Figure 2:**
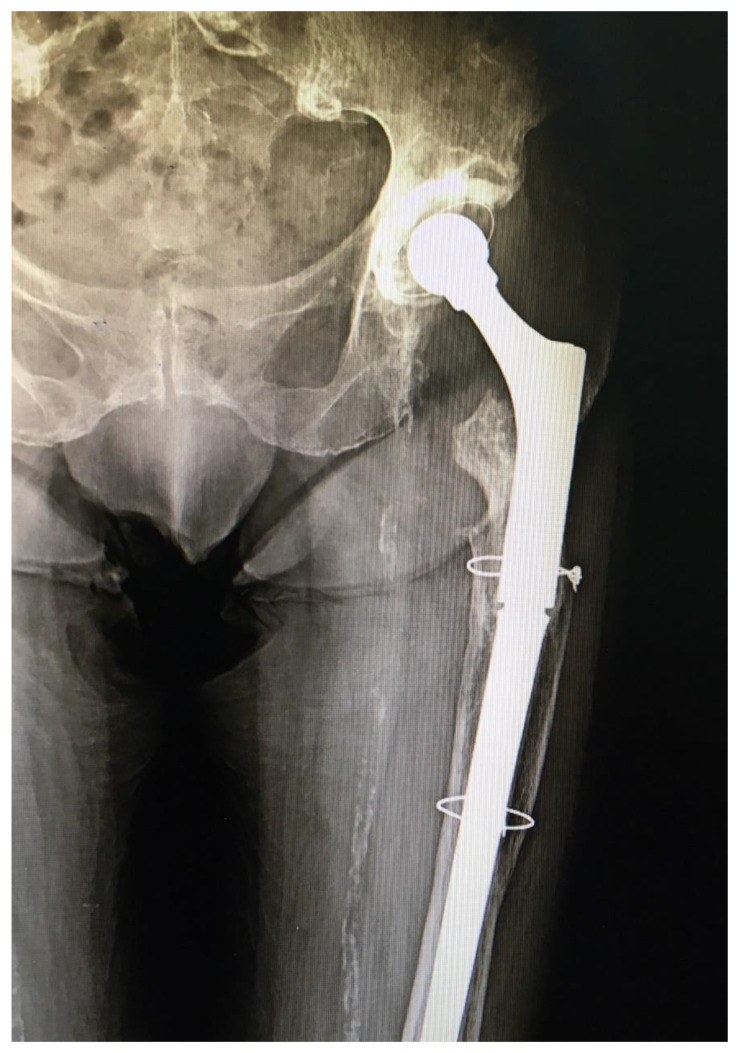
X-ray of the operated hip ten months after the surgery, showing a good position of the revision total hip endoprosthesis and no signs of instability.

The patient was stable throughout the duration of the surgery, with a total blood loss of 300 mL. On the second postoperative day, the patient started physical therapy, and she was verticalized with the walker. There were no perioperative complications and two and half years after surgery the patient is feeling well, has no pain in the operated hip, and walks with a walker because of her poor general condition. She has had no hip dislocations since this last operation. Postoperative follow-up X-rays show a stable hip endoprosthesis (Figure 2).

## 3. Discussion

Porous coating of acetabular cups improves bony in-growth and stability and reduces the risk of loosening [8,9]. However, there are rare cases of porous coating debonding that can complicate revision surgery. In the cases described in the literature, debonding happened much earlier than in our case [2,4]. Biomet Romanus acetabular component has been developed to combine the advantages of threaded and press-fit acetabular cups. Self-tapping threads were restricted to the periphery of the shell to give initial fixation, while the dome was coated with plasma-sprayed titanium porous coating allowing fixation by bone in-growth. Such porous coating has been considered mechanically strong because of chemical bonding of the metallic layers. In our case, the same type of implant was initially used, though the acetabular component was stable for more than twenty years. Von Knock [10] suggested three possible reasons for shedding of beads of porous coating: cup impaction, chemical corrosion, and micromotion. In our patient, osteoporosis due to her age and particle disease due polyethylene wear led to loosening of bone stock and micro-instability of acetabular component. Polyethylene wear is also possible reason of chemical corrosion between the porous coating and acetabular component. All these factors together with three falls by the patient could contribute to debonding of firmly ingrown porous coating from acetabular component.

According to Eskelinen’s [11] follow-up study from the Finnish arthroplasty register, Biomet Romanus cup also had problems with liner wear, osteolysis, and loosening, and enhanced acetabular components have been introduced since the late 1990s.

Additional problems in our case were the facts that the patient was older, in weak overall health, extremely osteoporotic, and had very poor acetabular bone stock. According to the literature, aseptic revision THA is associated with greater risks in patients aged ≥ 80 years compared with younger patients. Revision surgery is commonly associated with increased blood loss; loss of bone stock; intraoperative bone fracture; a higher risk of perioperative morbidity such as pneumonia, urinary tract infection, and the requirement for a blood transfusion; and an extended length of stay in hospital in patients aged ≥ 80 years [1]. Complex procedures of this sort can even end fatally [1]. Elderly patients with many comorbidities are weaker and they usually cannot unload operated leg, and for that reason, revision THA should provide primary stable components of endoprosthesis to allow early verticalization. Therefore, because most patients with poor overall health require only a painless joint with basic functionality for daily activity, a simpler and less risky operation should be the method of choice.

We do not think that porous coating left in acetabulum can pose any risk for the patient. It is a material that is well-fixed, osteo-integrated in the bone, and covered with bone cement. It is not in contact with the femoral head and cannot produce any particle wear, and for that reason, it should not cause any systemic response.

## 4. Conclusions

Acetabular component porous coating debonding is a rare complication in revision THA. Removal of the debonded layer is not a difficult procedure if there is normal bone stock of the acetabulum. In older patients with many comorbidities and osteoporotic bone stock of the acetabulum, revision surgery with a fixed debonded porous coating of the acetabular component can be challenging. In such a patient, revision procedure should be simplified and made as safe as possible. Leaving behind a well-fixed porous coating to serve as a “cage” to allow the cemented acetabular component placement and to reduce a blood loss could be a valuable option.
